# Platelet-activating factor (PAF) strongly enhances contractile mechanical activities in guinea pig and mouse urinary bladder

**DOI:** 10.1038/s41598-022-06535-7

**Published:** 2022-02-17

**Authors:** Ge Liu, Mizuki Kaneko, Kento Yoshioka, Keisuke Obara, Yoshio Tanaka

**Affiliations:** Department of Chemical Pharmacology, Faculty of Pharmaceutical Sciences, Toho University, Miyama 2-2-1, Funabashi, Chiba 274-8510 Japan

**Keywords:** Receptor pharmacology, Bladder

## Abstract

In this study, we investigated the effects of platelet-activating factor (PAF) on the basal tone and spontaneous contractile activities of guinea pig (GP) and mouse urinary bladder (UB) smooth muscle (UBSM) tissues to determine whether PAF could induce UBSM tissue contraction. In addition, we examined the mRNA expression of the PAF receptor, PAF-synthesizing enzyme (lysophosphatidylcholine acyltransferase, LPCAT), and PAF-degrading enzyme (PAF acetylhydrolase, PAF-AH) in GP and mouse UB tissues using RT-qPCR. PAF (10^−9^–10^−6^ M) strongly enhanced the basal tone and spontaneous contractile activities (amplitude and frequency) of GP and mouse UBSM tissues in a concentration-dependent manner. The enhancing effects of PAF (10^−6^ M) on both GP and mouse UBSM contractile activities were strongly suppressed by pretreatment with apafant (a PAF receptor antagonist, GP: 10^−5^ M; mouse: 3 × 10^−5^ M). The PAF receptor (*Ptafr*), LPCAT (*Lpcat1*, *Lpcat2*), and PAF-AH (*Pafah1b3*, *Pafah2*) mRNAs were detected in GP and mouse UB tissues. These findings reveal that PAF strongly enhances the contractile mechanical activities of UBSM tissues through its receptor and suggest that the PAF-synthesizing and -degrading system exists in UBSM tissues. PAF may serve as both an endogenous UBSM constrictor and an endogenous mediator leading to detrusor overactivity.

## Introduction

Platelet-activating factor (PAF) is a powerful mediator of inflammation and allergies. PAF was first discovered as a platelet agglutinin in 1972^1^, and its chemical structure was determined to be acetyl glyceryl ether phosphorylcholine in 1980^2^. In vivo and in vitro experiments clarified that PAF induces not only the aggregation and activation of platelets and leukocytes but also many various biological activities such as increasing vascular permeability, reducing cardiac output, and lowering blood pressure^3^. Furthermore, since the 1980s, PAF has been reported to be able to relax or contract various smooth muscle (SM) tissues. For example, (1) PAF relaxes rat thoracic/pulmonary/mesenteric artery SMs and ferret pulmonary artery in an endothelium-dependent manner^4–7^, and (2) PAF contracts human/rat/guinea pig (GP) tracheal and bronchial SMs^8–10^, rat gastric fundus SM^11^, GP ileal SM^12,13^, rat colonic SM^14^, and pregnant human/rat/GP uterine SM^15–17^. Thus, PAF exerts contractile effects on SMs of the respiratory, digestive, and genital organs, as well as relaxing effects on SMs of vessels in an endothelium-dependent manner.

PAF is produced not only in inflammatory cells such as eosinophils and neutrophils^18^ but also in cardiomyocytes, vascular endothelial cells, and urothelial cells that are not directly involved in inflammation^19–22^. Recently, the accumulation of PAF caused by smoking was reported in urinary bladder (UB) microvascular endothelial cells and urothelial cells, which suggests that PAF plays an important role in the development of chronic inflammatory UB diseases, such as interstitial cystitis and UB pain syndrome^21,22^. Additionally, the accumulation of PAF was significantly higher in isolated urothelial tissues from bladder cancer patients than in normal human urothelial tissues, and PAF expression was markedly increased in high-grade tumors compared with low-grade tumors^23^. These findings suggest that increased PAF production in urothelial cells is associated with lower urinary tract diseases. However, to the best of our knowledge, the effects of PAF on UBSM contractile mechanical activities have not been examined to date.

In this study, we investigated the effects of PAF on the basal tone and spontaneous contractile activities of UBSM tissues to determine whether PAF enhances the contractile mechanical activities of UBSMs. To confirm that the effect of PAF occurs irrespective of animal species, we used GPs, which exhibit similar lower urinary tract anatomy and urodynamic profile of micturition to those of humans^24^, and mice, for which exist various disease models. We also examined mRNA expression of the PAF receptor, PAF-synthesizing enzyme [lysophosphatidylcholine acyltransferase (LPCAT)], and PAF-degrading enzyme [PAF acetylhydrolase (PAF-AH)] in GP and mouse UB tissues to further understand whether PAF acts as an endogenous regulator of UBSM mechanical activities.

## Results

### Effects of PAF on GP UBSM basal tone and spontaneous contractile activities

**Figure 1:**
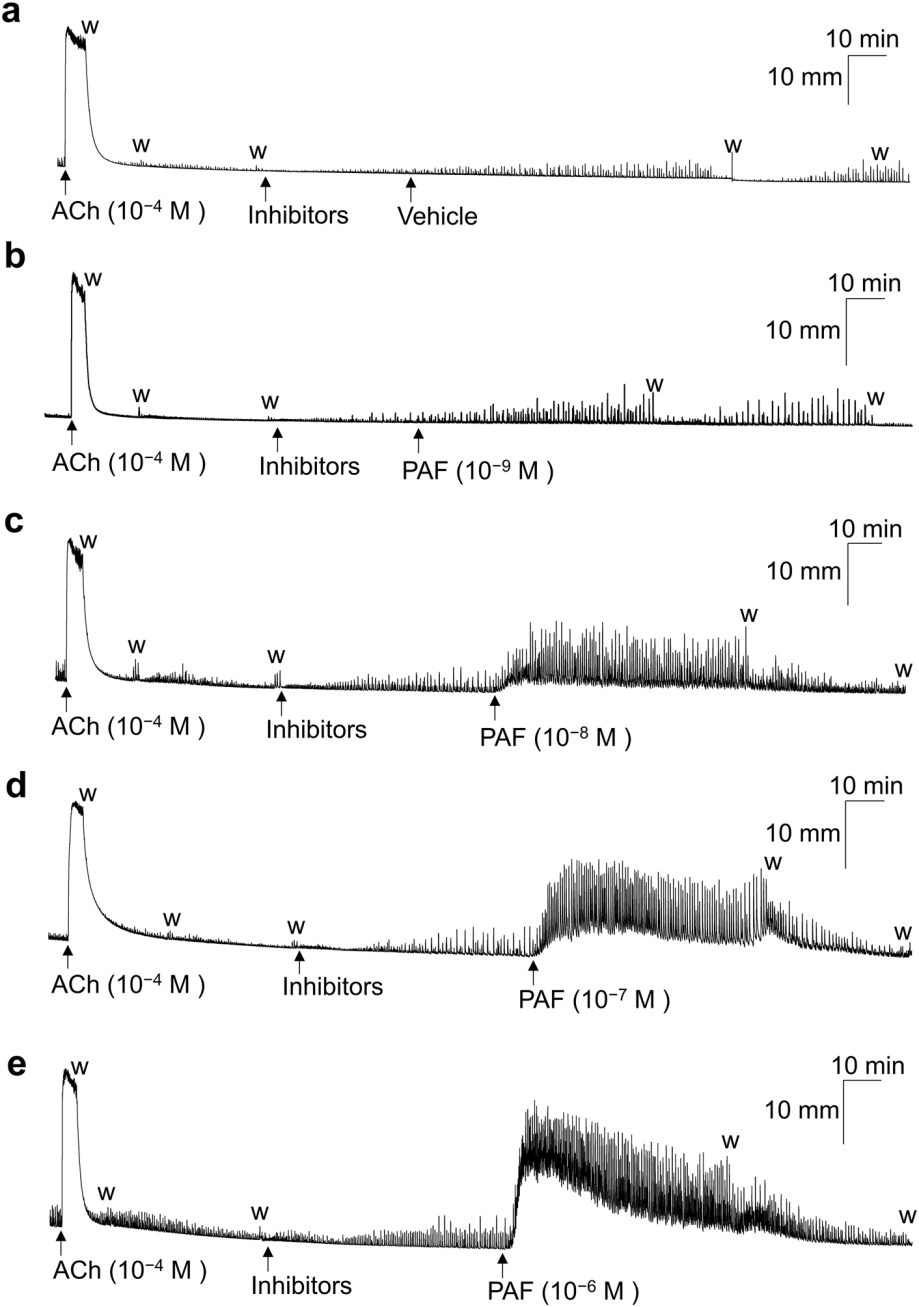
Representative traces showing the contractile response of acetylcholine (ACh, 10^−4^ M) and the effects of PAF [10^−9^ M (**b**); 10^−8^ M (**c**); 10^−7^ M (**d**); 10^−6^ M (**e**)] and its vehicle [0.25% bovine serum albumin (BSA) (**a**)] on the basal tone and spontaneous contraction activities in isolated guinea pig urinary bladder smooth muscle. Inhibitors: atropine (10^−6^ M), suramin (10^−4^ M), phentolamine (10^−6^ M), propranolol (10^−6^ M), tetrodotoxin (3 × 10^−7^ M), anti-foam (0.5%), and BSA (0.25%). *w* wash out, *PAF* platelet-activating factor.

**Figure 2:**
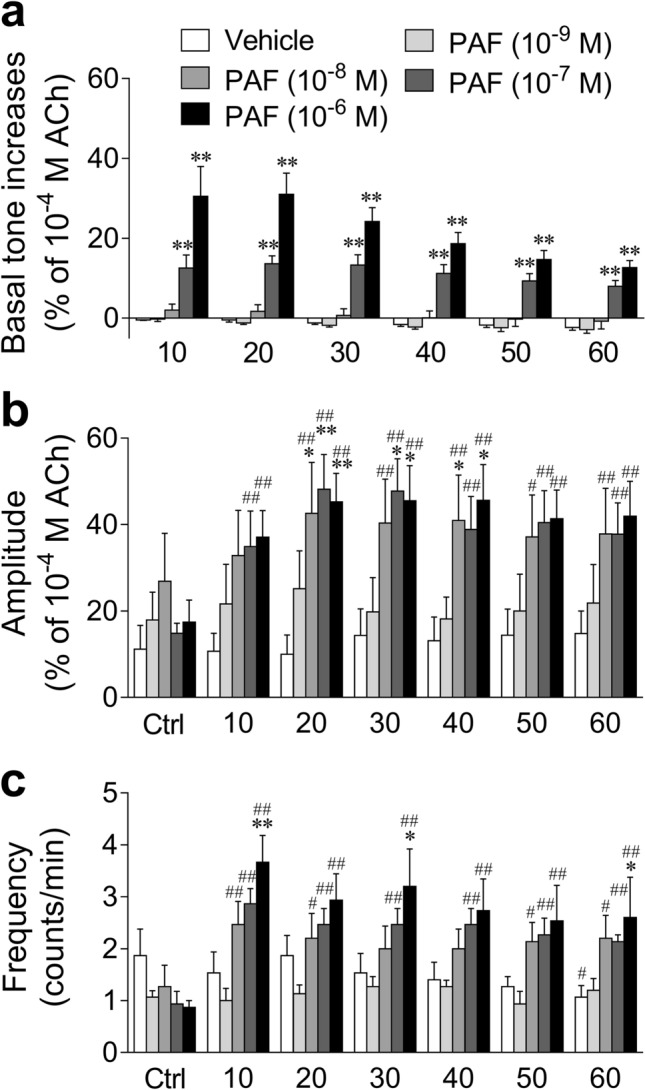
Summarized data of the effect of PAF (10^−9^–10^−6^ M) and its vehicle (0.25% bovine serum albumin) on the basal tone (**a**) and amplitude (**b**)/frequency (**c**) of spontaneous contractions in the isolated guinea pig urinary bladder smooth muscle shown in Fig. 1. Basal tone increases show basal tone changes before and after administration of PAF or its vehicle (**a**). Spontaneous contraction activities analyzed over 3 min during the following periods were calculated: immediately before administration of PAF or its vehicle (Ctrl, control); 7–10 min (10), 17–20 min (20), 27–30 min (30), 37–40 min (40), 47–50 min (50), and 57–60 min (60) after administration of PAF or its vehicle (**b**,**c**). Data are expressed as the means ± SEM (each *n* = 5). *P < 0.05; **P < 0.01 vs*.* the corresponding vehicle value; ^#^*P* < 0.05; ^##^*P* < 0.01 vs*.* the corresponding control value (two-way ANOVA followed by Dunnett’s test). *PAF* platelet-activating factor, *ACh* acetylcholine.

Figure 1 and 2 show typical experimental traces (Fig. 1) and quantified results (Fig. 2) of the effects of PAF (10^−9^–10^−6^ M) and its vehicle [0.25% bovine serum albumin (BSA)] on GP UBSM basal tone (Fig. 2a) as well as the amplitude (Fig. 2b) and frequency (Fig. 2c) of spontaneous contractile activities. To exclude the possible involvement of parasympathetic nerve- and sympathetic nerve-derived neurotransmitters [acetylcholine (ACh), ATP, and noradrenaline (NA)], a muscarinic receptor antagonist [atropine (10^−6^ M)], purine P2X receptor antagonist [suramin (10^−4^ M)], adrenoceptor antagonists [phentolamine (10^−6^ M) and propranolol (10^−6^ M)], and Na^+^ channel inhibitor [tetrodotoxin (TTX) (3 × 10^−7^ M)] were included in the experiment. The PAF vehicle (0.25% BSA) did not substantially affect the basal tone and spontaneous contractile activities of the GP UBSM (Figs. 1a, 2). In contrast, PAF (10^−9^–10^−6^ M) was found to increase GP UBSM mechanical activities in a concentration-dependent manner (Figs. 1b–e, 2). GP UBSM basal tone was remarkably increased by PAF at 10^−7^ M and 10^−6^ M. However, this enhancing effect on GP UBSM basal tone persisted for < 60 min and tended to start decreasing ~ 20–30 min after PAF administration (Figs. 1d,e, 2a). In contrast, the enhancing effects of PAF on GP UBSM spontaneous contractile activities were sustained for 60 min (Figs. 1, 2b,c). In addition, when the experiment shown in Figs. 1 and 2 (isotonic recording) was performed using the isometric recording (Supplementary Figs. 1 and 2), PAF strongly enhanced both basal tone and spontaneous GP UBSM mechanical activities.

### Effects of PAF on mouse UBSM basal tone and spontaneous contractile activities

**Figure 3:**
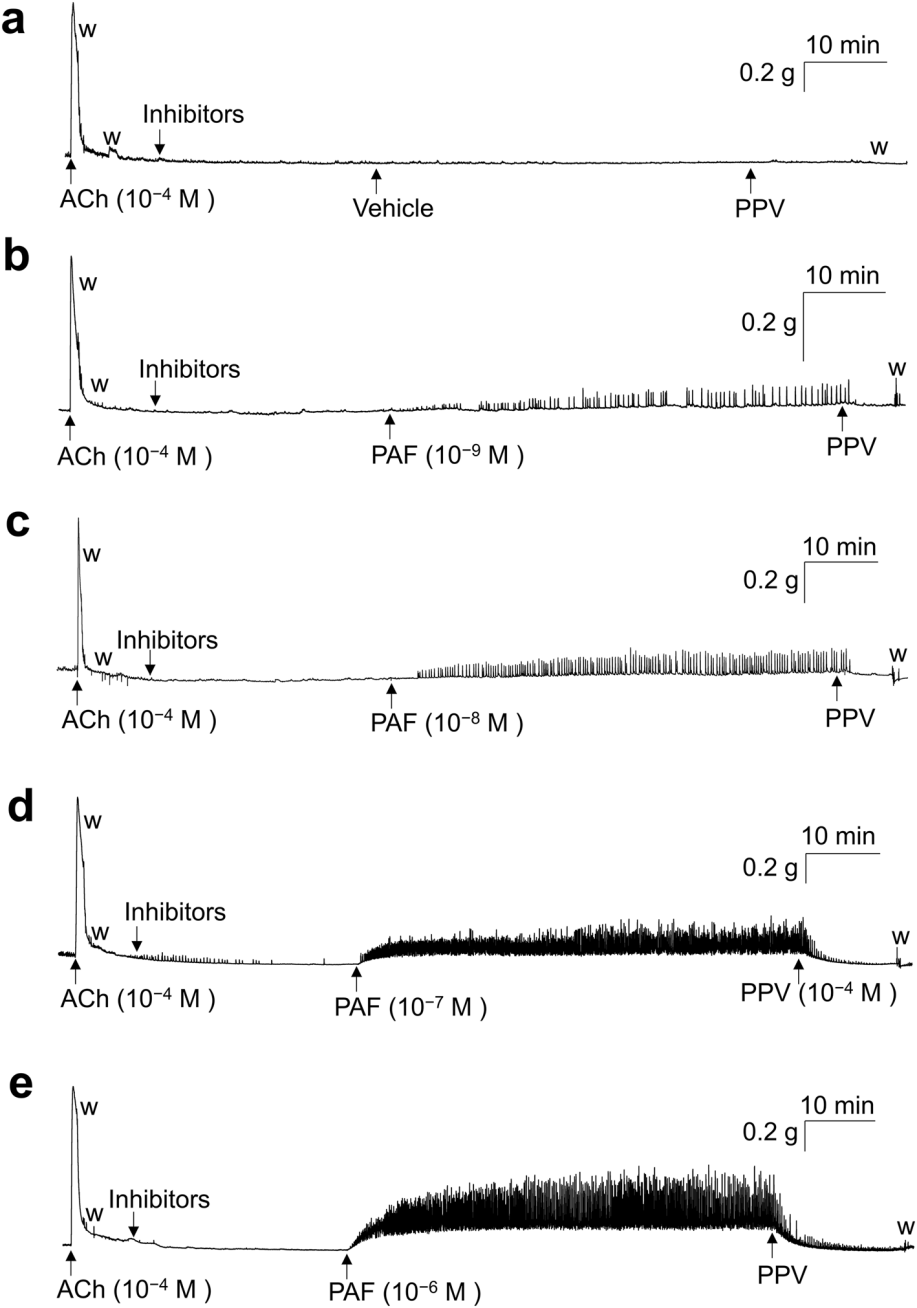
Representative traces showing the contractile response of acetylcholine (ACh, 10^−4^ M) and the effects of PAF [10^−9^ M (**b**); 10^−8^ M (**c**); 10^−7^ M (**d**); 10^−6^ M (**e**)] and its vehicle [0.25% bovine serum albumin (BSA) (**a**)] on the basal tone and spontaneous contraction activities in isolated mouse urinary bladder smooth muscle. Inhibitors: atropine (10^−6^ M), suramin (10^−4^ M), phentolamine (10^−6^ M), propranolol (10^−6^ M), tetrodotoxin (3 × 10^−7^ M), anti-foam (0.5%), and BSA (0.25%). *PPV* papaverine (10^−4^ M), *w* wash out, *PAF* platelet-activating factor.

**Figure 4:**
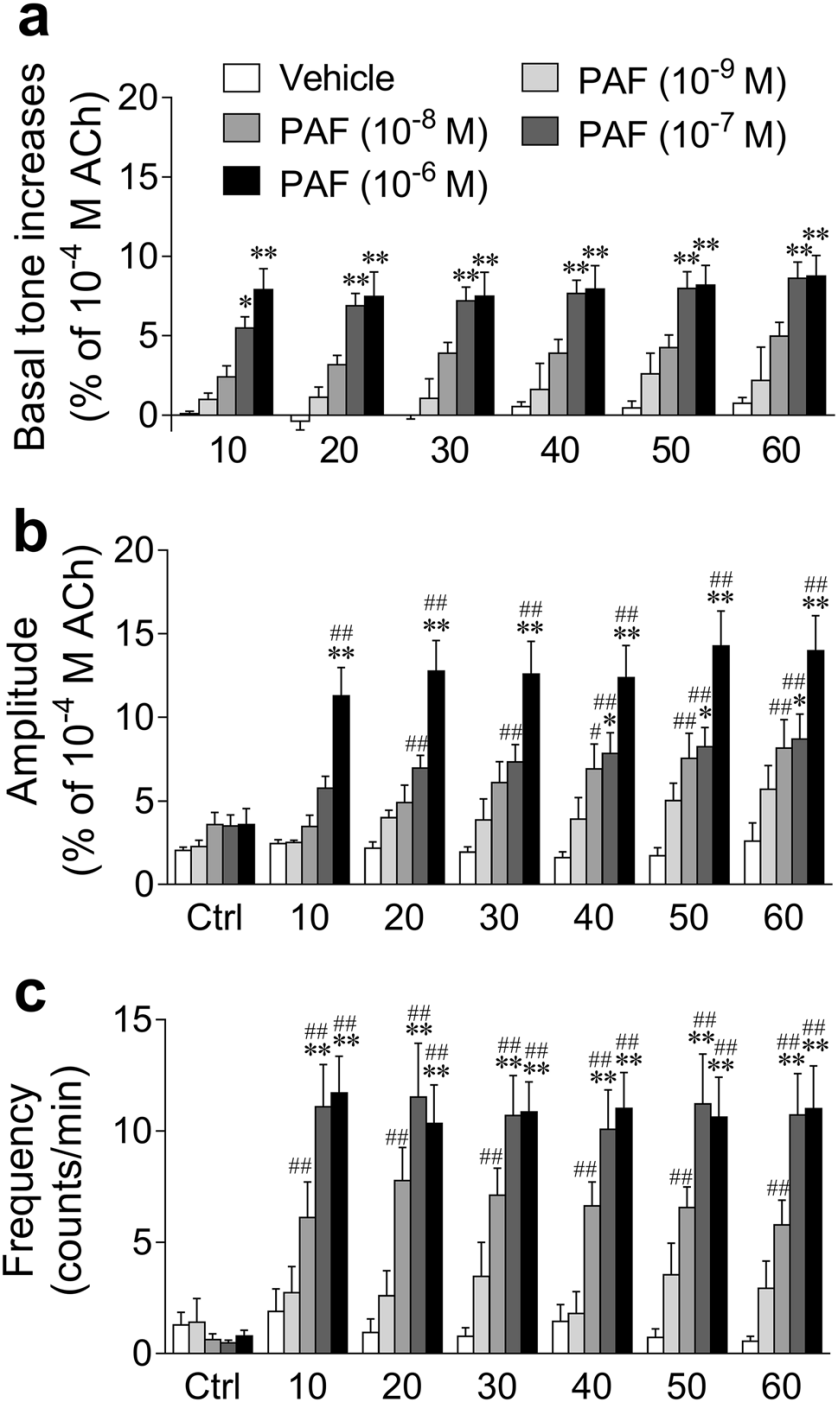
Summarized data of the effect of PAF (10^−9^–10^−6^ M) and its vehicle (0.25% bovine serum albumin) on the basal tone (**a**) and amplitude (**b**)/frequency (**c**) of spontaneous contractions in the isolated mouse urinary bladder smooth muscle shown in Fig. 3. Basal tone increases show basal tone changes before and after administration of PAF or its vehicle (**a**). Spontaneous contraction activities analyzed over 3 min during the following periods were calculated: immediately before administration of PAF or its vehicle (Ctrl, control); 7–10 min (10), 17–20 min (20), 27–30 min (30), 37–40 min (40), 47–50 min (50), and 57–60 min (60) after administration of PAF or its vehicle (**b**,**c**). Data are expressed as the means ± SEM [*n* = 6 (vehicle), *n* = 5 (10^−9^ M), *n* = 9 (10^−8^ M), *n* = 14 (10^−7^ M), *n* = 16 (10^−6^ M)]. *P < 0.05; **P < 0.01 vs*.* the corresponding vehicle value; ^#^*P* < 0.05; ^##^*P* < 0.01 vs. the corresponding control value (two-way ANOVA followed by Dunnett’s test). *PAF* platelet-activating factor, *ACh* acetylcholine.

Figures 3 and 4 show typical experimental traces (Fig. 3) and quantified results (Fig. 4) of the effects of PAF (10^−9^–10^−6^ M) and its vehicle (0.25% BSA) on mouse UBSM basal tone (Fig. 4a), as well as the amplitude (Fig. 4b) and frequency (Fig. 4c) of spontaneous contractile activities. This study was also carried out in the presence of the antagonists and inhibitors described in the previous section. The PAF vehicle (0.25% BSA) did not substantially affect basal tone and spontaneous contractile activities of mouse UBSM (Figs. 3a, 4). In contrast, PAF (10^−9^–10^−6^ M) was found to increase mouse UBSM mechanical activities in a concentration-dependent manner (Figs. 3b–e, 4). The enhancement of mouse UBSM mechanical activities was sustained for 60 min (Figs. 3, 4).

### Effects of apafant on UBSM basal tone and spontaneous contractile activities enhanced by PAF

**Figure 5:**
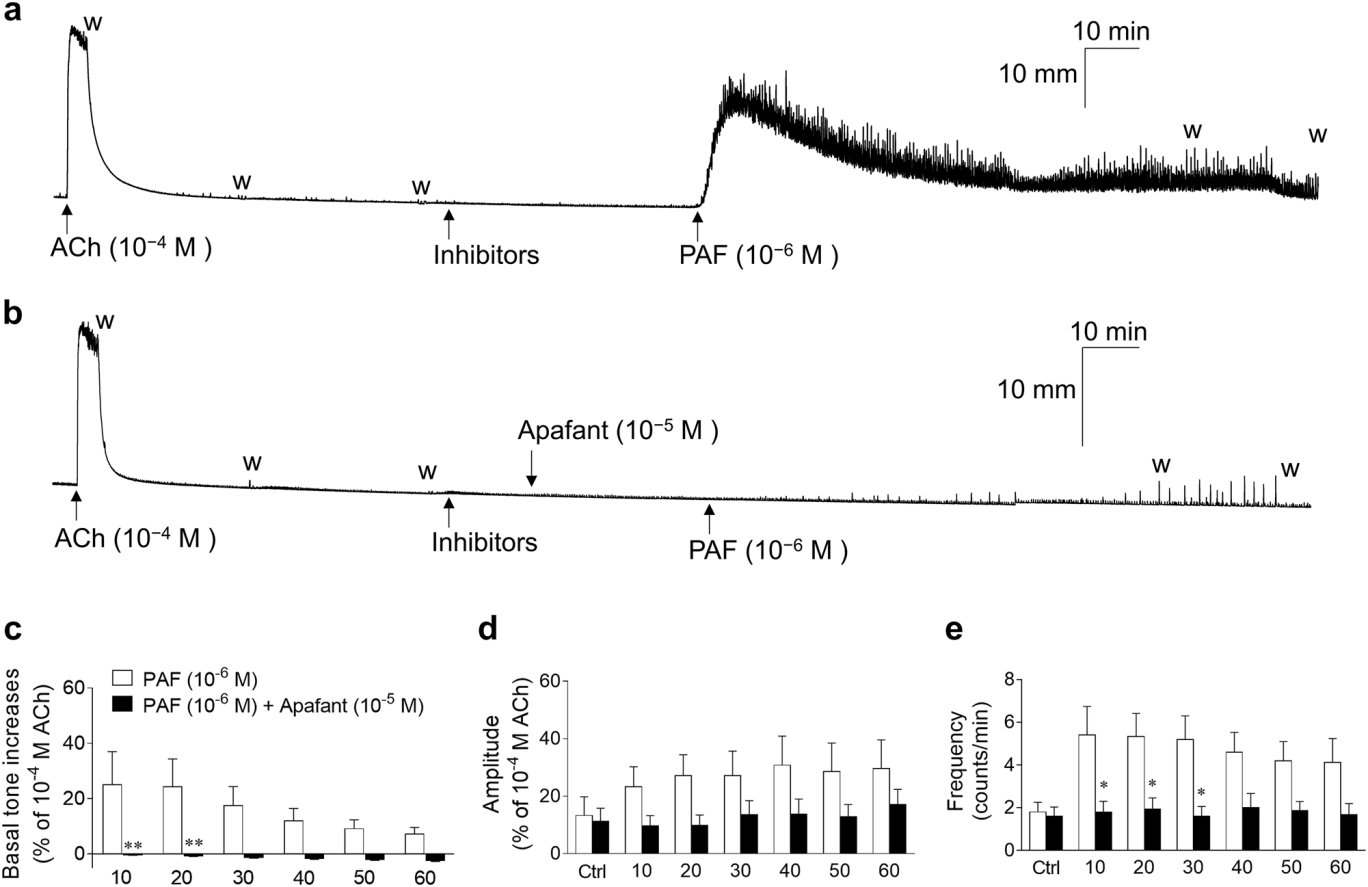
Representative traces (**a**,**b**) and summarized data (**c**–**e**) showing the effects of pretreatment of apafant (10^−5^ M, **b**) on guinea pig urinary bladder smooth muscle basal tone increases (**c**) and amplitude (**d**)/frequency (**e**) of spontaneous contraction activities enhanced by PAF (10^–6^ M). Basal tone increases show the basal tone changes before and after administration of PAF (**c**). Spontaneous contraction activities analyzed over 3 min during the following periods were calculated: immediately before administration of PAF (Ctrl, control); 7–10 min (10), 17–20 min (20), 27–30 min (30), 37–40 min (40), 47–50 min (50), and 57–60 min (60) after administration of PAF (**d**,**e**). Data are expressed as the means ± SEM (each *n* = 5). *P < 0.05; **P < 0.01 vs. the corresponding PAF alone value (multiple *t-*tests). Inhibitors: atropine (10^−6^ M), suramin (10^−4^ M), phentolamine (10^−6^ M), propranolol (10^−6^ M), tetrodotoxin (3 × 10^−7^ M), anti-foam (0.5%), and bovine serum albumin (0.25%). *w* wash out, *PAF* platelet-activating factor, *ACh* acetylcholine.

Figure 5 shows typical experimental traces (Fig. 5a,b) and quantified results (Fig. 5c–e) of the effects of apafant (a PAF receptor antagonist, 10^−5^ M) on GP UBSM basal tone (Fig. 5c), as well as the amplitude (Fig. 5d) and frequency (Fig. 5e) of spontaneous contractile activities enhanced by PAF (10^−6^ M). The enhancing effects of PAF (10^−6^ M) on GP UBSM basal tone (Fig. 5c) and the amplitude (Fig. 5d) and frequency (Fig. 5e) of spontaneous contractile activities were almost completely suppressed by apafant (10^−5^ M) (Fig. 5b–e).

**Figure 6:**
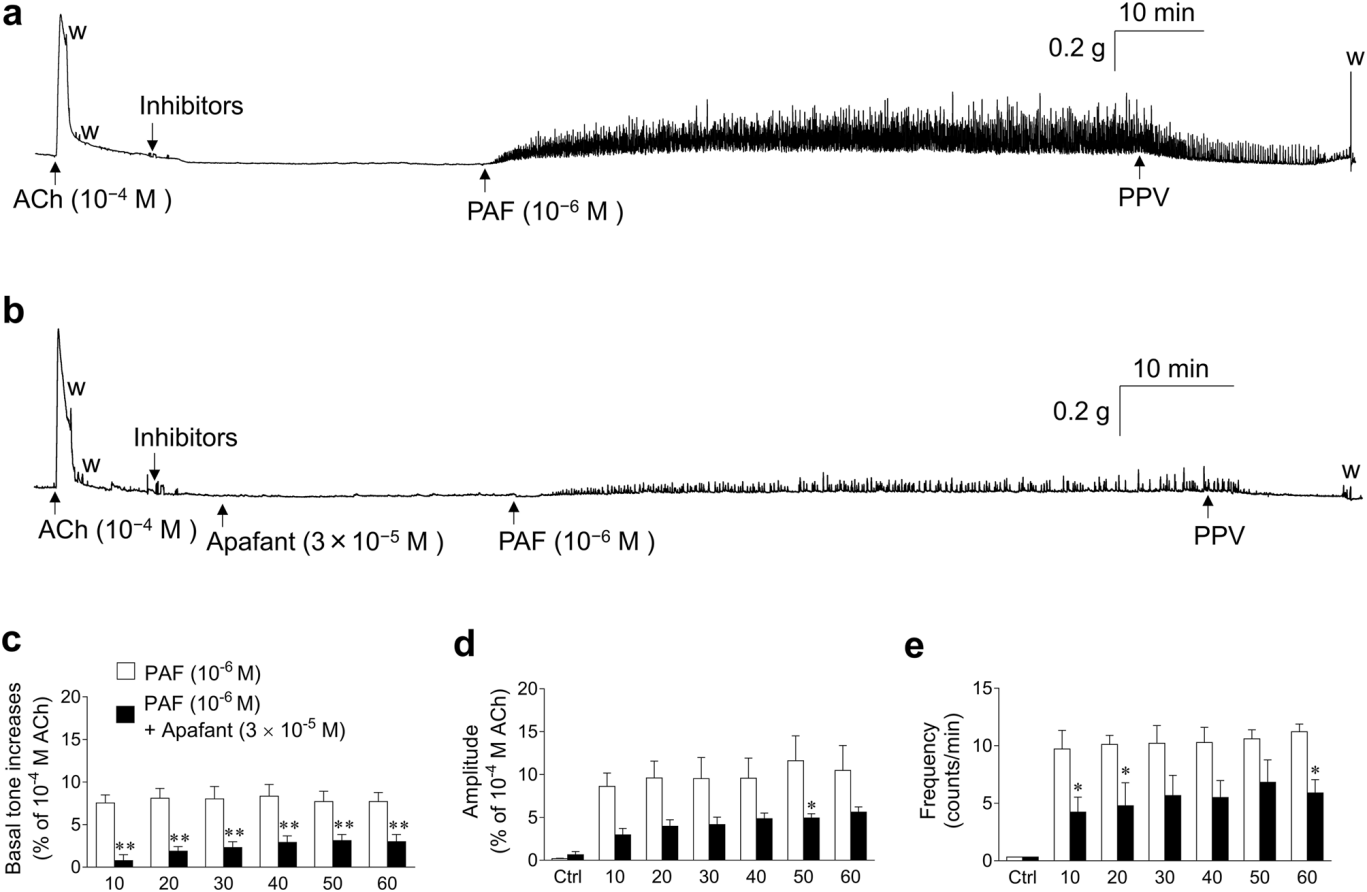
Representative traces (**a**,**b**) and summarized data (**c**–**e**) showing the effects of pretreatment of apafant (3 × 10^−5^ M, **b**) on mouse urinary bladder smooth muscle basal tone increases (**c**) and amplitude (**d**)/frequency (**e**) of spontaneous contraction activities enhanced by PAF (10^–6^ M). Basal tone increases show the basal tone changes before and after administration of PAF (**c**). Spontaneous contraction activities analyzed over 3 min during the following periods were calculated: immediately before administration of PAF (Ctrl, control); 7–10 min (10), 17–20 min (20), 27–30 min (30), 37–40 min (40), 47–50 min (50), and 57–60 min (60) after administration of PAF (**d**,**e**). Data are expressed as the means ± SEM (each *n* = 5). *P < 0.05; **P < 0.01 vs. the corresponding PAF alone value (multiple *t-*tests). Inhibitors: atropine (10^−6^ M), suramin (10^−4^ M), phentolamine (10^−6^ M), propranolol (10^−6^ M), tetrodotoxin (3 × 10^−7^ M), anti-foam (0.5%), and bovine serum albumin (0.25%). *PPV* papaverine (10^−4^ M), *w* wash out, *PAF* platelet-activating factor, *ACh* acetylcholine.

Figure 6 shows typical experimental traces (Fig. 6a,b) and quantified results (Fig. 6c–e) of the effects of apafant (3 × 10^−5^ M) on mouse UBSM basal tone (Fig. 6c), as well as the amplitude (Fig. 6d) and frequency (Fig. 6e) of spontaneous contractile activities enhanced by PAF (10^−6^ M). The enhancing effects of PAF (10^−6^ M) on mouse UBSM basal tone (Fig. 6c) and the amplitude (Fig. 6d) and frequency (Fig. 6e) of spontaneous contractile activities were very strongly suppressed by apafant (3 × 10^−5^ M).

### mRNA expressions of PAF receptor (*Ptafr*), LPCAT (*Lpcat1, Lpcat2*), and PAF-AH (*Pafah1b3, Pafah2*) in GP and mouse UB tissues

**Figure 7:**
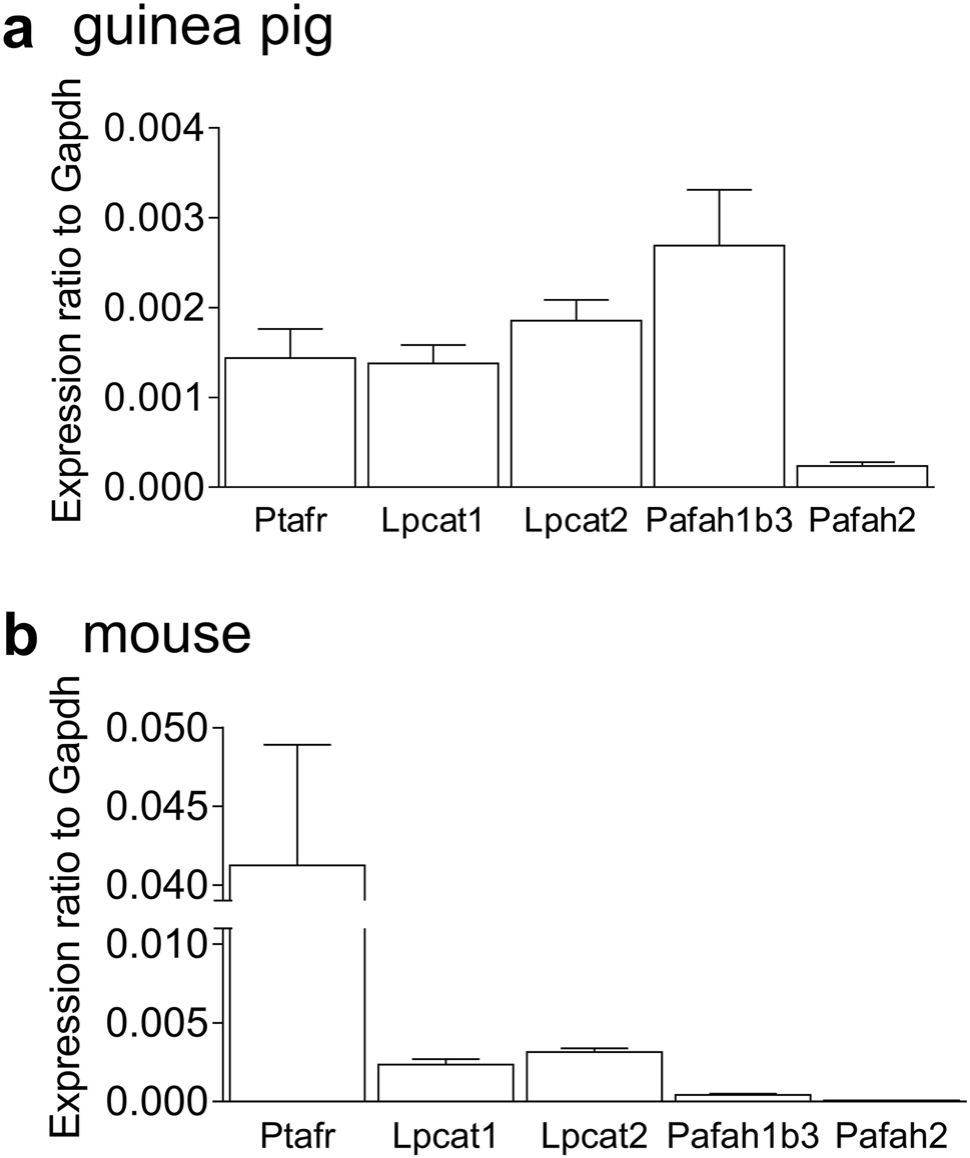
mRNA expression levels of PAF receptor (*Ptafr*), PAF-synthesizing enzymes (*Lpcat1, Lpcat2*), and PAF-degrading enzymes (*Pafah1b3, Pafah2*) in guinea pig (**a**) and mouse (**b**) urinary bladder tissues as assessed by RT-qPCR. The expression level of each mRNA is shown relative to the mRNA expression level of *Gapdh*, which is arbitrarily set as 1. Data are expressed as the means ± SEM [*n* = 8 (**a**) and *n* = 7–10 (**b**)]. *PAF* platelet-activating factor.

Figure 7 shows the relative mRNA expressions of PAF receptor (*Ptafr*), LPCAT (*Lpcat1* and *Lpcat2*), and PAF-AH (*Pafah1b3* and *Pafah2*) in GP (Fig. 7a) and mouse (Fig. 7b) UB tissues as assessed by quantitative reverse transcription PCR (RT-qPCR). All mRNA expression levels have been normalized to that of *Gapdh*. *Ptafr* is expressed in both GP and mouse UB tissues. LPCAT mRNA (*Lpcat1, Lpcat2*) and PAF-AH mRNA (*Pafah1b3, Pafah2*) are also expressed in GP and mouse UB tissues; however, the expression of *Pafah2* is extremely low compared to that of *Pafah1b3*.

## Discussion

In this study, we found that PAF (10^−9^–10^−6^ M) strongly enhanced the basal tone and spontaneous contractile activities (amplitude and frequency) of GP and mouse UBSM tissues in a concentration-dependent manner. The enhancing effects of PAF (10^−6^ M) on both UBSM contractile activities were very strongly suppressed by apafant pretreatment (GP: 10^−5^ M; mouse: 3 × 10^−5^ M). PAF receptor (*Ptafr*), LPCAT (*Lpcat1* and *Lpcat2*), and PAF-AH (*Pafah1b3* and *Pafah2*) mRNAs were found to be expressed in GP and mouse UB tissues. These findings reveal that PAF strongly enhances contractile mechanical activities of UBSM tissues through the PAF receptor and suggest that the PAF-synthesizing and PAF-degrading system may exist in UBSM tissues. PAF may be both an endogenous UBSM constrictor and an endogenous mediator leading to detrusor overactivity.

ACh and ATP are the best-known endogenous UBSM constrictors. These neurotransmitters are released from nerve endings of the parasympathetic nervous system that innervates UBSM, and they play a physiologically important role in the UB micturition reflex. In fact, ACh and ATP are involved in the contractile responses induced by electrical field stimulation in isolated GP and mouse UBSM tissues^25,26^. Tachykinins (neurokinin A and substance P) are also known as endogenous UBSM contractile substances^27^. Although these substances induce contraction of isolated UBSM tissues, they are considered to promote the UB micturition reflex physiologically by affecting the central nervous system^27^. Thus, the peripheral physiological roles of tachykinins on UBSM contractile functions are still unclear. In recent years, prostanoids have also attracted attention as endogenous UBSM constrictors. Specifically, prostaglandin (PG) A_2_, PGE_2_, PGF_2α_, and thromboxane A_2_ strongly induce UBSM contractions and enhance spontaneous contractile activities^28^. PAF was reported to contract various SM tissues (tracheal and bronchial SMs^8–10^, uterine SMs from pregnant females^15–17^, and gastrointestinal SMs^11–14^). In this study, we found that PAF is able to potently contract isolated GP and mouse UBSM tissues and enhance their mechanical contractile activities. To the best of our knowledge, ours is the first report of the effect of PAF on UBSM contractile functions.

PAF was first identified as a substance produced and released from inflammatory cells^1^. Subsequent research revealed that PAF is synthesized not only by inflammatory cells but also by cells that are not directly involved in inflammation, such as cardiomyocytes and endothelial cells^18–20^. Interestingly, most cells that synthesize PAF also express the PAF receptor^18^. The biosynthetic routes of PAF involve two different pathways, the remodeling pathway and the de novo pathway^3,29^. The remodeling pathway is a route that converts cell membrane lipid into the PAF precursor lyso-PAF. Specifically, alkylacyl phosphatidylcholines, a subclass of membrane phospholipids, are metabolized to lyso-PAF by phospholipase A_2_ (PLA_2_), and lyso-PAF is then converted to PAF via LPCAT^3,29^. In contrast, the de novo pathway is a route that converts ether lipid intermediates into PAF. It consists of three steps: (1) acetylation/acylation, (2) dephosphorylation, and (3) transfer of the phosphocholine moiety from CDP-choline to a diradylgycerol via alkylacetylglycerol dithiothreitol-insensitive cholinephosphotransferase^3,29^. After stimulating its target cells, PAF is rapidly hydrolyzed to biologically inactive lyso-PAF by PAF-AH, a PLA_2_ enzyme^3,29^. The remodeling pathway appears to be the main mechanism of PAF synthesis in stimulated neutrophils, monocytes, and eosinophils when inflammatory and allergic disorders occur, and the de novo pathway may play a role in the homeostatic functions of organs such as the brain and kidney^3,29–32^. In contrast, in endothelial cells, PAF is reported to be biosynthesized via the remodeling pathway^3^.

PAF increased the mechanical activity of GP UBSM more strongly than that of mouse UBSM. GP is often used as a research model for the human urinary tract, as its lower urinary tract anatomy and urodynamic profile of micturition are similar to those of humans^24^. Additionally, GP UB contains the contractile muscularis mucosae (SM) in addition to the detrusor SM, similar to the human UB, whereas mouse UB is deficient in the contractile muscularis mucosae^33^. Therefore, in GP UB, PAF could contract not only the detrusor SM but also muscularis mucosae, leading to a stronger increase in mechanical activity. However, mouse UB appeared to express higher levels of PAF receptor mRNA compared with GP UB. The appearance of stronger contractions in GP UB with lower PAF receptor mRNA expression compared with mouse UB with higher PAF receptor mRNA expression was not consistent with the general assumption that there is a correlation between the receptor mRNA expression levels and changes in SM contractile function. A reason for this phenomenon might be that the PAF-induced contractile response in mouse UBSM can be strongly affected by the suppressing factors. For example, the spontaneous contractile activity of the mouse bladder is considerably weaker than that of the GP UBSM (Figs. 1 and 3), and the large conductance calcium-activated K^+^ channel (BK channel) was reported to strongly suppress the mouse UBSM spontaneous contraction^34^. Therefore, the PAF-induced contractile response might also be strongly suppressed by the BK channel; nevertheless, this hypothesis warrants further clarification in the future.

In GP UBSM, the enhancing effect of PAF (10^−6^ M) on basal tone persisted for < 60 min and tended to start decreasing ~ 20–30 min after PAF administration. This concentration of PAF may strongly stimulate the PAF receptor in GP UBSM, resulting in desensitization. Reportedly, repeated administration of PAF diminishes the contraction response induced by PAF in rat stomach SM^11^. Inflammation induced by infusion of trinitrobenzene sulfonic acid in GP ileum has also been reported to cause desensitization of PAF receptors. Desensitization of PAF receptors in the ileum depends on the duration of inflammation and correlates with PAF content in the ileum, suggesting that PAF receptor desensitization may play a protective role by preventing overstimulation of intestinal SM^35^. Therefore, in GP UBSM, excessive PAF stimulation might cause PAF receptor desensitization, which may have a protective effect on the UBSM. In addition, the mRNA expression level of the PAF-degrading enzyme (PAF-AH) was higher in the GP UB than in the mouse UB (Fig. 7). Therefore, another reason why the enhancing effect of PAF (10^−6^ M) in GP UBSM did not persist may be that PAF is more likely to be degraded in GP UB than in mouse UB.

PAF was reported to increase intracellular Ca^2+^ concentration in rat mesenteric vein and human uterine smooth muscle cells^36,37^. In human uterine smooth muscle cells, inositol trisphosphate was involved in the PAF-induced increase in intracellular Ca^2+^ concentration^37^. In rat stomach smooth muscles, the contractile response induced by PAF depends on Ca^2+^ influx from the extracellular fluid, wherein the underlying mechanism involves the nicardipine-sensitive Ca^2+^ influx pathway (voltage-gated Ca^2+^ channel)^11^; this pathway involved in the PAF-induced contractile responses of UBSM warrants further investigation.

Recently, the connection between PAF and chronic UB inflammatory responses caused by smoking has been reported. Exposure to cigarette smoke results in (1) increased PAF production and decreased PAF-AH activity in human and mouse UB microvascular endothelial cells^21^ and (2) increased PAF production and PAF receptor expression in human urothelial cells^22^. In addition, calcium-independent PLA_2_ (iPLA_2_) was reported to catalyze PAF biosynthesis in both UB microvascular endothelial cells and urothelial cells^21,22^. Due to long-term cigarette smoke exposure, both PAF expression and PAF receptor expression were increased in the UB of wild-type mice, whereas neither was detected in the UB of iPLA_2_ (iPLA_2_β)-knockout mice^22^. In the present study, we detected the mRNA expression of PAF receptor (*Ptafr*), LPCAT (*Lpcat1* and *Lpcat2*), and PAF-AH (*Pafah1b3* and *Pafah2*) in both GP and mouse UB tissues. Our findings suggest that PAF is involved in the regulation of physiological contractile functions in both GP and mouse UB tissues, although it still cannot be concluded whether the UB epithelium, UBSM, or other cells express the abovementioned mRNA. This should be clarified in future studies. However, the following evidence suggests that PAF receptors are present at least in the GP UBSM: (1) the enhancing effects of PAF (10^−6^ M) on contractile activities were almost identical in epithelial-intact and epithelial-removed GP UB tissues (Supplementary Fig. 3) and (2) PAF receptor mRNA expression was also detected in epithelial-removed GP UB tissues (Supplementary Fig. 4).

As mentioned above, PAF may be involved in the pathogenesis of chronic inflammatory UB diseases caused by smoking, such as interstitial cystitis and bladder pain syndrome^21,22^. PAF may also be involved in bladder cancer caused by smoking; for example, PAF accumulation was significantly higher in isolated urothelial tissues from UB cancer patients than in normal human urothelial tissues, and PAF expression was markedly higher in high-grade tumors than in low-grade tumors^23^. Furthermore, the association between smoking and overactive bladder (OAB) has recently been reported. Namely, (1) the relative risk of OAB in males was higher for ex-smokers and current smokers than for non-smokers^38^, and (2) cigarette smoking is associated with OAB in females^39^. Thus, PAF might also be involved in the OAB caused by smoking. From this point of view, we highlight the potential role of PAF receptor antagonists as new therapeutic agents for the abovementioned smoking-caused UB diseases.

## Methods

### Animals

Male GPs (age 5–8 weeks old; weight 310–500 g; Kyudo Co., Ltd., Saga, Japan) and male mice (age 9–16 weeks old; weight 38–56 g; Japan SLC, Hamamatsu, Japan) were housed under controlled conditions (20–22°C, relative air humidity 50 ± 5%) and a fixed 12/12 h light/dark cycle (08:00–20:00), with food and water available ad libitum. This study was approved by the Toho University Animal Care and Use Committee (approval number: 21-52-444) and was conducted in accordance with the guidelines of the Laboratory Animal Center of Faculty of Pharmaceutical Sciences, Toho University. This study was carried out in compliance with the ARRIVE guidelines.

### Preparation of UBSM strips

GPs and mice were anesthetized using isoflurane inhalation and euthanized by exsanguination via the carotid artery. Thereafter, the UBs of GPs/mice were rapidly excised and immersed in modified/normal Locke–Ringer solution containing (mM) NaCl, 154; KCl, 5.63; CaCl_2_, 1.2/2.16; MgCl_2_, 2.1; NaHCO_3_, 5.95; glucose, 2.78. After removing surrounding adipose and connective tissues and the UB trigone, the UB was opened with a longitudinal incision, and UBSM strips (GP: approximately 2 mm in width × 20 mm in length; mouse: approximately 1 mm in width × 10 mm in length) were prepared. UB epithelial cells were not removed in this study unless otherwise stated.

### Measurement of UBSM basal tone and spontaneous contractile activities

The GP and mouse UBSM strips were suspended under a resting tone of 1.0 g and resting tension of 0.5 g, respectively, in a 20 mL organ bath filled with modified/normal Locke–Ringer solution at 32 ± 1.0°C and bubbled with a mixture of 95% O_2_ and 5% CO_2_. GP UBSM length changes were isotonically recorded with isotonic transducers and amplifiers (IT-300/IT-AM-300, Physio-Tech Co., Ltd., Tokyo, Japan), and mouse UBSM tension changes were isometrically recorded with isometric transducers and amplifiers (TB-612T/AP-620G, Nihon Kohden, Tokyo, Japan). All data were recorded using PowerLab™ and LabChart™ (Version 7) software (ADInstruments Pty. Ltd., Bella Vista, NSW, Australia).

The UBSM strips were equilibrated for ≥ 20 min and then contracted using ACh (10^−4^ M) at least 3 times at 10 min intervals. After UBSM basal tone stabilized, phentolamine (10^−6^ M, an α-adrenoceptor antagonist), propranolol (10^−6^ M, a β-adrenoceptor antagonist), atropine (10^−6^ M, a muscarinic receptor antagonist), suramin (10^−4^ M, a purine P2X receptor antagonist), TTX (3 × 10^−7^ M, a Na^+^ channel inhibitor), anti-foam (0.5%), and BSA (0.25%) were administered, and the strips were incubated for 30 min. This inhibitor cocktail was used to eliminate the possible effects of peripheral nerve-derived neurotransmitters. As shown in Supplementary Fig. 5, the contractions induced by ACh (3 × 10^−6^ M) were completely suppressed by atropine (10^−6^ M) in both GP (Supplementary Fig. 5a) and mouse (Supplementary Fig. 5e) UBSMs. Moreover, the relaxations induced by NA (10^−6^ M) in the presence of bethanechol (3 × 10^−6^ M) and phentolamine (10^−6^ M) were completely suppressed by propranolol (10^−6^ M) (Supplementary Fig. 5d,h). In addition, the contractions induced by ATP (10^−4^ M/3 × 10^−4^ M) were strongly suppressed by suramin (10^−4^ M) (Supplementary Fig. 5b,f). Since NA (10^−6^ M) and phenylephrine (10^−6^ M, a selective α_1_-adrenoceptor agonist) did not contract GP or mouse UBSMs in the presence of propranolol (10^−6^ M) (Supplementary Fig. 5c,g), substantial involvement of α_1_-adrenoceptor in both UBSMs was considered negligible. Phentolamine (10^−6^ M) was reported to strongly suppress the NA-induced blood vessel contractions^40^. We previously confirmed that TTX almost completely eliminates ACh- and ATP-mediated contractile responses elicited by electrical field stimulations in both GP and mouse UBSMs^25,26^.

All the experiments were performed in the presence of indomethacin (3 × 10^−6^ M, a cyclooxygenase inhibitor) to eliminate the effects of endogenous prostanoids.

### Effects of PAF on GP and mouse UBSM basal tone and spontaneous contractile activities

After the procedures described in the previous section, PAF (10^−9^–10^−6^ M) or its vehicle (0.25% BSA) was administered in the bath medium, and the strips were incubated for 60 min. Afterward, the GP UBSM strips were washed with modified Locke–Ringer solution, and the mouse UBSM strips were relaxed with papaverine (PPV, 10^–4^ M). Reportedly, PPV induces the contractile response in GP UBSM^41^. Herein, we confirmed that PPV enhanced spontaneous contractile activity and did not suppress the contractile response by PAF, as shown in Supplementary Fig. 1. Therefore, we performed the wash-out operation without using PPV in GP UBSM.

### Effects of apafant on UBSM basal tone and spontaneous contractile activities enhanced by PAF

Apafant (a PAF receptor antagonist, GP: 10^−5^ M; mouse: 3 × 10^−5^ M) was administered in the bath medium concomitantly with the inhibitor cocktail described in “Measurement of UBSM basal tone and spontaneous contractile activities”. Afterward, the effects of PAF (10^−6^ M) were determined as described in “Effects of PAF on GP and mouse UBSM basal tone and spontaneous contractile activities”.

### RT-qPCR of mRNA expression of PAF receptor (*Ptafr*), LPCAT (*Lpcat1, Lpcat2*), and PAF-AH (*Pafah1b3, Pafah2*)

Total RNA was extracted from isolated GP and mouse UBs using the acid guanidinium thiocyanate-phenol–chloroform method^42^. The extracted total RNA was treated with deoxyribonuclease (Nippon Gene Co. Ltd., Tokyo, Japan) at 37°C for 30 min. Phenol–chloroform extraction was performed after contaminating DNA removal, followed by ethanol precipitation. The RNA pellets were dissolved in diethyl pyrocarbonate-treated water. First-strand cDNA was synthesized by reverse transcription with 1 μg total RNA per 20 μL reaction mixture using the ReverTra Ace^®^ qPCR RT Master Mix with gDNA Remover (TOYOBO Co., Ltd., Osaka, Japan) according to the manufacturer's protocol.

RT-qPCR was performed using the THUNDERBIRD^®^ Next SYBR^®^ qPCR Mix (TOYOBO Co. Ltd.) according to the manufacturer's protocol. The primers used in this study are shown in Table 1. PCR and DNA amplification (fluorescence intensity) measurements were performed using a 7500 Fast Real-Time PCR System (Applied Biosystems, Waltham, MA, USA). The thermal cycler parameters were set at 95°C for 2 min, followed by 40 cycles of denaturation at 95°C for 15 s, annealing at 60°C for 30 s, and elongation at 72°C for 35 s. DNA amplification (fluorescence intensity) was measured at each elongation step. After PCR completion, the melting curves of each PCR product were measured from 60–95°C. The data were analyzed using Sequence Detection Software Version 1.4 (Applied Biosystems). Samples that did not reach the fluorescence intensity threshold after 40 cycles of amplification were considered to have no detectable mRNA expression. The mRNA expression level of each gene was calculated as a relative value, normalized to the mRNA expression level of the glyceraldehyde 3-phosphate dehydrogenase gene (*Gapdh*), which was set to 1.

**Table 1:** Primers used for RT-qPCR.

Animal species	Gene symbol	Sequence (5ʹ–3ʹ)
Guinea pig	*Ptafr*	GAAGAAGCCGAGCACAATGC
		ATCAAGACTGCTCAGGCCAC
Guinea pig	*Lpcat1*	TGATTGCCCTGTCAGTCGTG
		CCAGAGCGGTCCTGAGAATG
Guinea pig	*Lpcat2*	ACGTTGCCTATGGAAGCTGG
		TCGAGGCACTGGCAATAGAC
Guinea pig	*Pafah1b3*	ACCCACTCTCGGCTTTTCAG
		ACGACTTCGGGTTCCTTGTC
Guinea pig	*Pafah2*	TCCTGGGTGGTTTCTCATTTCC
		CTGGCTGGTCCCCATCTTATC
Guinea pig	*Gapdh*	ACGGATTTGGCCGTATTGGA
		CCATTCTCAGCCTTGACGGT
Mouse	*Ptafr*	CGAGGGCGACTGGATTCTAC
		CAAAGAGATGCCACGCTTGC
Mouse	*Lpcat1*	GGACCTGGAGCGTTGAAAATC
		CATGACACGCCTCACATTGC
Mouse	*Lpcat2*	TGGCCCCAGATACTAGTTTTCC
		TATAGCCTTGCCAGGTCCAG
Mouse	*Pafah1b3*	CCGTCCACTCTGGGTTGTG
		CGCAGACTAGAAACCGTCAC
Mouse	*Pafah2*	TTCTGTGTTCTGTCTTCCAGCC
		CTGTACTCGTAGCGGGGAATC
Mouse	*Gapdh*	GATGACATCAAGAAGGTGGTGA
		TGCTGTAGCCGTATTCATTGTC

### Drugs

The following drugs were used in this study: PAF C-16 and apafant (Cayman Chemical, Ann Arbor, MI, USA); TTX, suramin sodium and anti-foam (FUJIFILM Wako Pure Chemical Co., Osaka, Japan.); phentolamine mesylate (Novartis Pharma KK, Tokyo, Japan); dl-propranolol hydrochloride, atropine sulfate salt monohydrate, and indomethacin (Sigma-Aldrich Co., St. Louis, MO, USA); and BSA (fatty acid free, pH 7.0; Nacalai Tesque Inc., Kyoto, Japan).

PAF was dissolved in ethanol (EtOH) to prepare a stock solution of 2 × 10^−3^ M and stored at −80°C. When using PAF, the EtOH solvent was evaporated, and PAF was redissolved and diluted with 0.25% BSA to prepare solutions of 2 × 10^−4^–2 × 10^−7^ M. Indomethacin was dissolved in EtOH to prepare a stock solution of 10^−2^ M. All other drugs were dissolved and diluted with distilled water.

### Data analysis

UBSM basal tone and amplitude/frequency of spontaneous contraction activities were analyzed using the cyclic measurement mode of LabChart 7, considering the minimum point of each spontaneous contraction event after PAF application as the baseline level. The minimum average amplitude (basal tone), average amplitude, and the total number of UBSM spontaneous contractile activities over 3 min during the following periods were calculated: immediately before administration of PAF (control) and 7–10 min, 17–20 min, 27–30 min, 37–40 min, 47–50 min, and 57–60 min after administration of PAF. For these analyses, the cutoff value was set at one third of the largest spontaneous contraction immediately before administration of PAF for 3 min (GP) or 1% of the third 10^−4^ M ACh-induced contraction described in “Measurement of UBSM basal tone and spontaneous contractile activities” (mouse). Basal tone increases show the basal tone changes before and after administrations of PAF. UBSM basal tone increases and amplitude of spontaneous contractile activities are shown as relative values, with the third 10^−4^ M ACh-induced contraction of the procedure described in “Measurement of UBSM basal tone and spontaneous contractile activities” set as 100%. The frequency of UBSM spontaneous contractile activities (counts/min) was calculated by dividing the total number of spontaneous contractile activities over 3 min by 3 min.

Data are expressed as the means ± standard error of the mean (SEM), where *n* refers to the number of experiments. Statistical analyses were carried out with two-way ANOVA followed by Dunnett’s tests or multiple *t*-tests using GraphPad Prism™ (Version 6) (GraphPad Software, Inc., San Diego, CA, USA). All statistical analyses were conducted with a significance level of α = 0.05 (*P* < 0.05).

## Supplementary Information

Supplementary Figures.

## Data Availability

The data that support the findings of this study are available from the corresponding author, KO, upon reasonable request.
