# Supplementary material for: Platelet-activating factor (PAF) strongly enhances contractile mechanical activities in guinea pig and mouse urinary bladder
Source: Sci Rep. 2022 Feb 17;12:2783. doi: 10.1038/s41598-022-06535-7 (PMC8854422; doi:10.1038/s41598-022-06535-7)
Supplement: Supplementary file 1 — Supplementary Figures. [file 41598_2022_6535_MOESM1_ESM.pdf]

Scientific reports / Article

Platelet-activating factor (PAF) strongly enhances contractile mechanical activities in guinea pig and mouse urinary bladder

Ge Liu, Mizuki Kaneko, Kento Yoshioka, Keisuke Obara*, Yoshio Tanaka

Department of Chemical Pharmacology, Faculty of Pharmaceutical Sciences, Toho University, Miyama 2-2-1, Funabashi-City, Chiba 274-8510, Japan

***Correspondence to:**
Keisuke Obara, Ph.D.
Department of Chemical Pharmacology
Faculty of Pharmaceutical Sciences, Toho University
Miyama 2-2-1, Funabashi-City
Chiba 274-8510, Japan.
E-mail: keisuke.obara@phar.toho-u.ac.jp

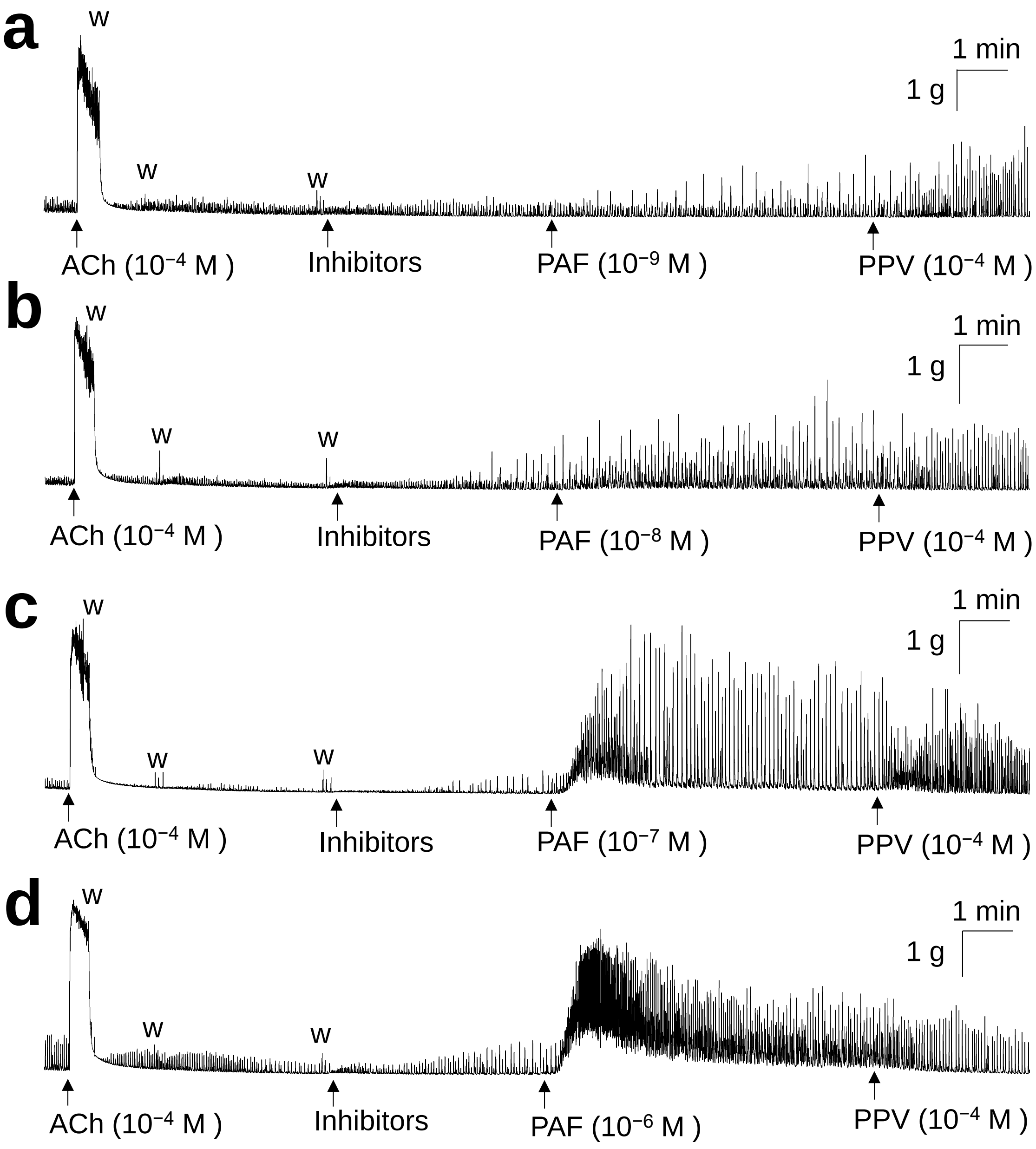

Supplementary Fig. 1

Isometric recoding of representative traces showing the contractile response of acetylcholine (ACh, 10^{-4} M) and the effects of PAF (10^{-9} M, **a**; 10^{-8} M, **b**; 10^{-7} M, **c**;10^{-6} M, **d**) and papaverine (PPV, 10^{-4} M) on the basal tone and spontaneous contraction activities in isolated guinea pig urinary bladder smooth muscle. Inhibitors: atropine (10^{-6} M), suramin (10^{-4} M), phentolamine (10^{-6} M), propranolol (10^{-6} M), tetrodotoxin (3×10^{-7} M), anti-foam (0.5%), and BSA (0.25%). w: wash out; PAF: platelet-activating factor.

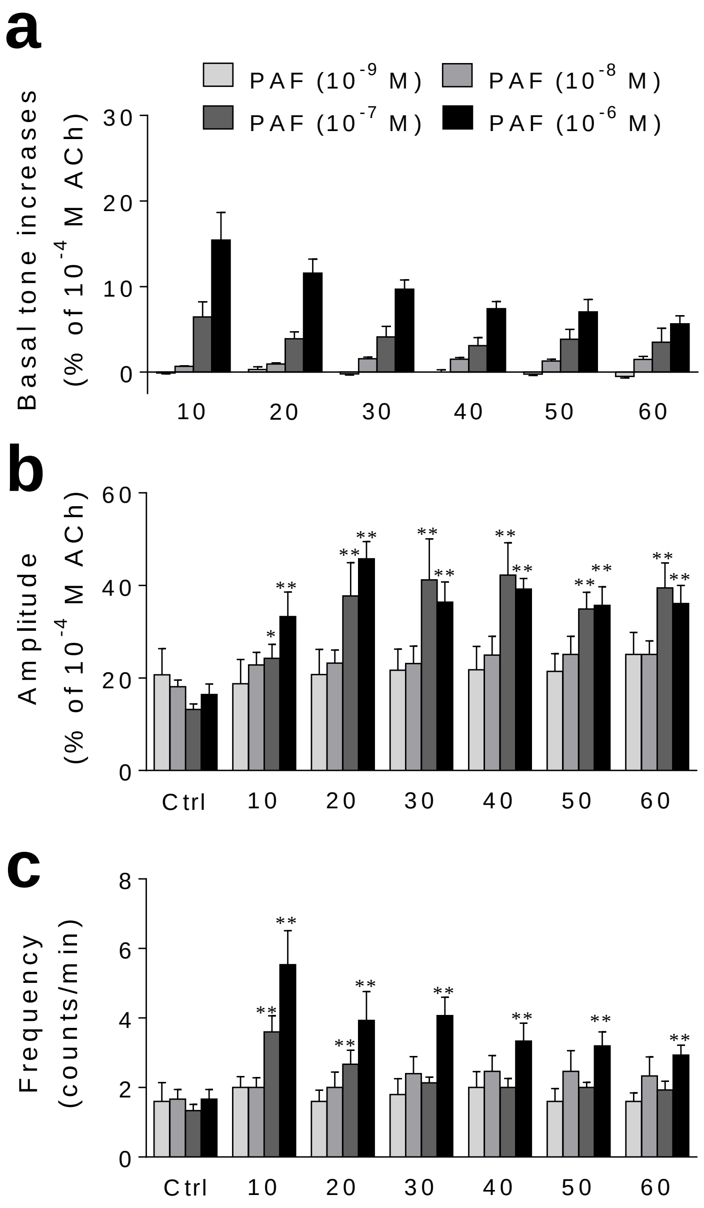

Supplementary Fig. 2

Summarized data of the effect of PAF (10^{-9}–10^{-6} M) on the basal tone (**a**) and amplitude (**b**)/frequency (**s**) of spontaneous contractions in the isolated guinea pig urinary bladder smooth muscle shown in **Supplementary Fig. 1**. Basal tone increases show basal tone changes before and after administration of PAF (**a**). Spontaneous contraction activities analyzed over 3 min during the following periods were calculated: immediately before administration of PAF (Ctrl, control); 7–10 min (10), 17–20 min (20), 27–30 min (30), 37–40 min (40), 47–50 min (50), and 57–60 min (60) after administration of PAF (**b, c**). Data are expressed as the means ± SEM (each n = 5). *$P < 0.05$; **$P < 0.01$ *vs.* the corresponding control value (two-way ANOVA followed by Dunnett's test). PAF: platelet-activating factor; ACh: acetylcholine.

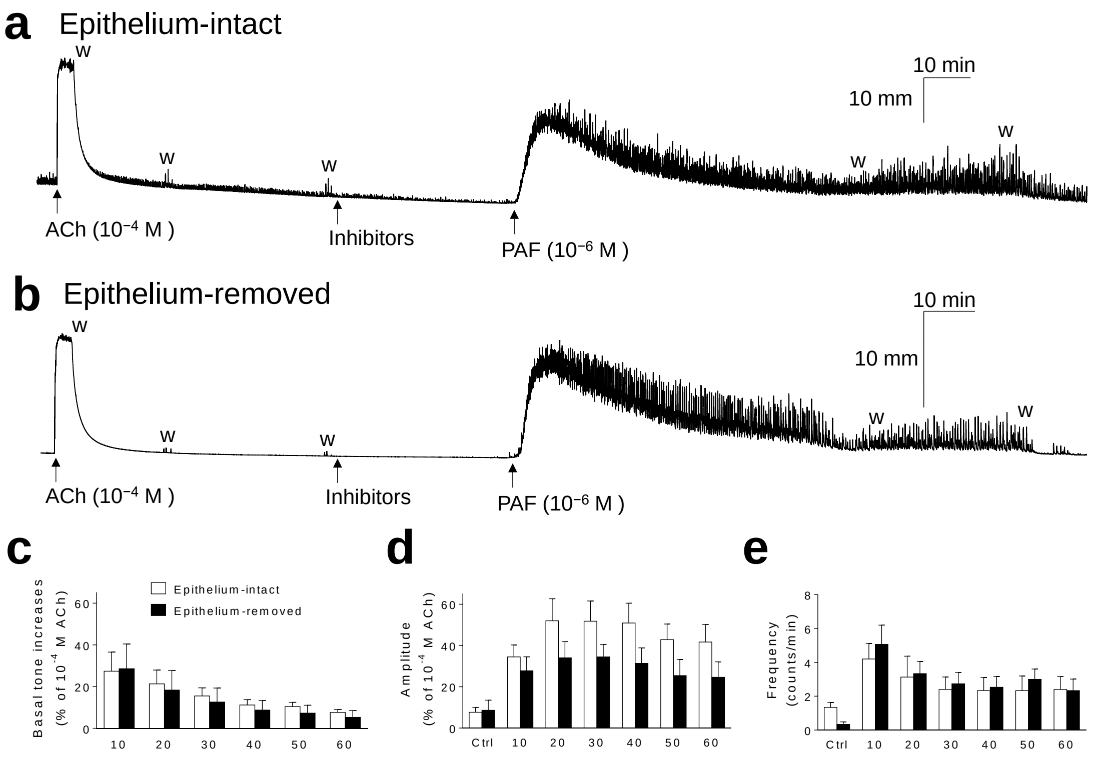

Supplementary Fig. 3

Representative traces (**a**, **b**) and summarized data (**c**–**e**) showing the effects of PAF (10^{-6} M) on guinea pig urinary bladder smooth muscle basal tone (**c**) and amplitude (**d**)/frequency (**e**) of spontaneous contraction activities in epithelium-intact (**a**) and epithelium-removed (**b**) preparations. Basal tone increases show the basal tone changes before and after administration of PAF (**c**). Spontaneous contraction activities analyzed over 3 min during the following periods were calculated: immediately before administration of PAF (Ctrl, control); 7–10 min (10), 17–20 min (20), 27–30 min (30), 37–40 min (40), 47–50 min (50), and 57–60 min (60) after administration of PAF (**d, e**). Data are expressed as the means ± SEM (each $n = 5$). Inhibitors: atropine (10^{-6} M), suramin (10^{-4} M), phentolamine (10^{-6} M), propranolol (10^{-6} M), tetrodotoxin (3×10^{-7} M), anti-foam (0.5%), and bovine serum albumin (0.25%). w: wash out; PAF: platelet-activating factor; ACh: acetylcholine.

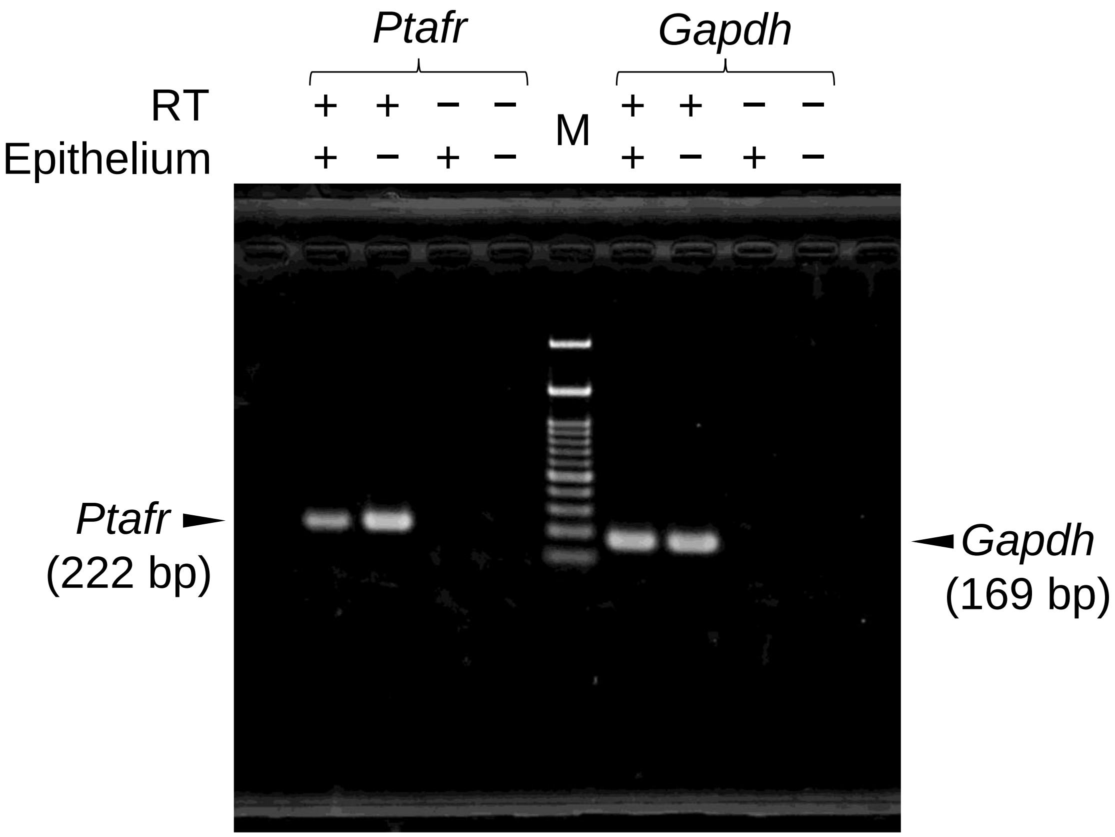

Supplementary Fig. 4

mRNA expression of PAF receptor (*Ptafr*) and *Gapdh* in guinea pig epithelium-intact (+) and epithelium-removed (−) urinary bladder tissues as assessed by RT-PCR. The primers used in this experiment are the same as shown in Table 1. PCR was performed with GoTaq® Green Master Mix (Promega Corp., Madison, WI, USA) and TaKaRa PCR Thermal Cycler Dice® Touch (TaKaRa Bio Inc., Shiga, Japan). PCR samples were heated for 2 min at 95°C and then amplified for 32 cycles (for *Ptafr*) or 26 cycles (for *Gapdh*) at 95°C for 15 s, 60°C for 20 s, and 72°C for 20 s followed by a 5-min extension at 72°C. The PCR products and FastGene 100bp DNA Ladder RTU (M) (NIPPON genetics, Tokyo, Japan) were separated by electrophoresis on a 2% agarose gel, stained with ethidium bromide (0.5 µg/mL) for 10 min, and visualized under UV illumination. RT: reverse transcription.

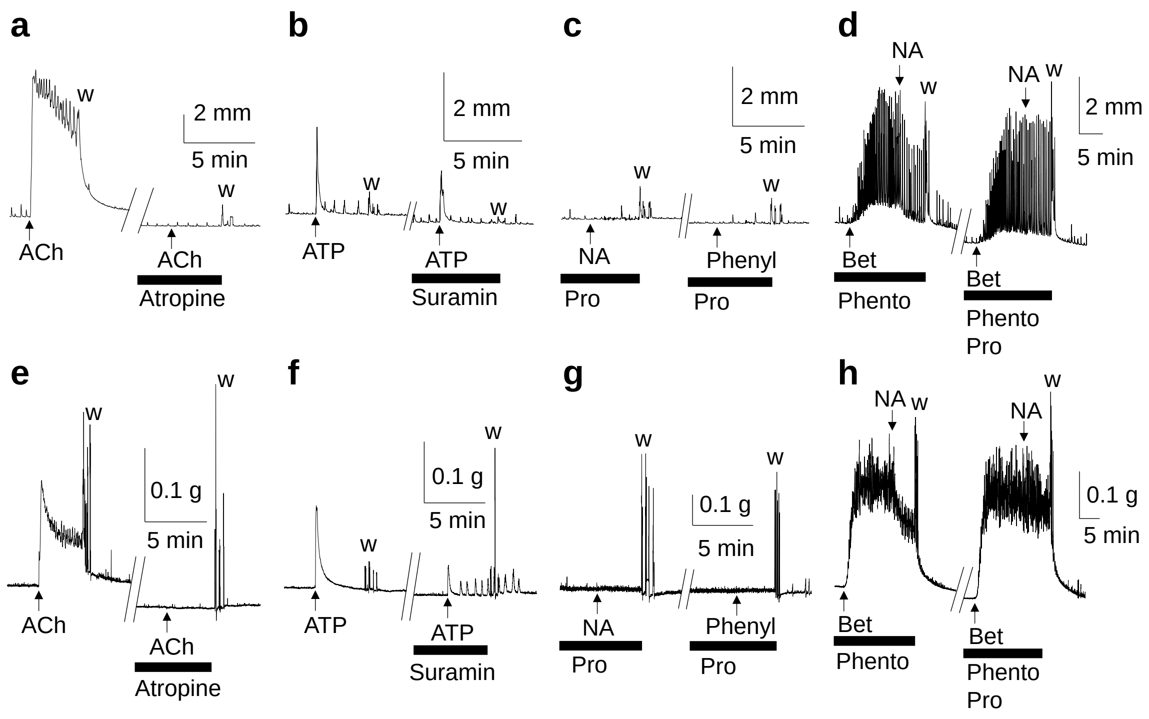

Supplementary Fig. 5

Representative traces showing the contractions (**a–c, e–g**)/relaxations (**d, h**) induced by acetylcholine (ACh, 3×10^{-6} M, **a, e**), ATP (10^{-4} M, **b**; 3×10^{-4} M, **f**), noradrenaline (NA, 10^{-6} M, **c, d, g, h**), and phenyrephrine (Phenyl, 10^{-6} M, **c, g**) and the effects of atropine (10^{-6} M, **a, e**), suramin (10^{-4} M, **b, f**), and propranolol (Pro, 10^{-6} M, **d, h**) on these contractions/relaxations in guinea pig (**a–d**) and mouse (**e–h**) urinary bladder smooth muscle. The contractions induced by NA and Phenyl were recorded in the presence of Pro (10^{-6} M, **c, g**). The relaxations induced by NA were recorded in the presence of bethanechol (Bet, 3×10^{-6} M) and phentolamine (Phento, 10^{-6} M) (**d, h**). w: wash out.
